# Complement C3c Reflects Acute-Phase Response but Not Clinical Phenotype in Systemic Sclerosis: A Cross-Sectional Study

**DOI:** 10.3390/ijms27135839

**Published:** 2026-06-28

**Authors:** Jakub Trefler, Anna Pasierb, Lidia Lech, Hubert Czaplicki, Katarzyna Życińska

**Affiliations:** 1 Department of Rheumatology, Connective Tissue Diseases, and Rare Diseases, National Medical Institute of the Ministry of the Interior and Administration, ul. Wołoska 137, 02-507 Warsaw, Poland; 2 Department of Family Medicine, Medical University of Warsaw, ul. Żwirki i Wigury 61, 02-091 Warsaw, Poland

**Keywords:** systemic sclerosis, complement system proteins, biomarkers, inflammation, autoimmune diseases, connective tissue diseases

## Abstract

This study evaluated routinely measured serum complement C3c (C3c) and complement C4 (C4) in relation to systemic inflammation, clinical and immunological phenotypes, and patient-reported outcomes (PROs) in systemic sclerosis (SSc). Seventy SSc patients fulfilling the 2013 American College of Rheumatology/European League Against Rheumatism (ACR/EULAR) criteria underwent same-day assessment including serum C3c, C4, C-reactive protein (CRP), erythrocyte sedimentation rate (ESR), interleukin-6 (IL-6), autoantibodies, immunosuppressive therapy and PROs. Associations were analysed using Spearman correlations and linear regression. C3c correlated strongly with CRP (rho = 0.53, *p* < 0.001) and ESR (rho = 0.49, *p* < 0.001) and remained independently associated with CRP (regression coefficient β = 0.26 mg/L per mg/dL, 95% confidence interval [CI] 0.14–0.38, *p* < 0.001) and ESR (β = 0.32 mm/h per mg/dL, 95% CI 0.16–0.47, *p* < 0.001), explaining ~17% and 15% of their variance. C4 showed weaker correlations with CRP (rho = 0.37, *p* = 0.004) and ESR (rho = 0.29, *p* = 0.03). Neither C3c nor C4 correlated with IL-6, modified Rodnan skin score (mRSS), interstitial lung disease (ILD), gastrointestinal involvement, SSc subset, autoantibodies, immunosuppressive therapy or PROs. C3c and C4 levels were significantly lower in patients with secondary Sjögren’s disease (SjD) than SSc-only. Serum C3c reflects acute-phase response in SSc, paralleling CRP and ESR but not clinical phenotype or patient-perceived burden, whereas C4 provides only weaker, secondary information.

## 1. Introduction

Systemic sclerosis (SSc) is a complex autoimmune connective tissue disease characterized by immune dysregulation, vasculopathy, and progressive fibrosis of the skin and internal organs. Although its clinical course is heterogeneous, inflammatory and immune-mediated mechanisms are increasingly recognized as key upstream drivers of tissue injury and disease progression. Identifying biologically meaningful and clinically interpretable biomarkers that reflect these processes remains a major challenge in SSc, particularly given the limited performance of traditional inflammatory markers in this disease [1,2,3].

The complement system is a central component of innate immunity and an important amplifier of inflammation and tissue damage in autoimmune diseases [4,5]. In systemic lupus erythematosus and primary SjD, serum complement measurements—particularly C3 and C4—are routinely used as biomarkers of immune complex-mediated activity and disease monitoring. In SSc, however, the role of complement is more complex and less clearly translated into clinical practice. Early studies demonstrated activation of the complement cascade in SSc and reported associations between complement consumption and disease severity [6]. Subsequent work identified distinct complement activation patterns in selected high-risk manifestations, such as scleroderma renal crisis [7], and large multicentre cohorts showed that hypocomplementemia in SSc is relatively uncommon and frequently associated with overlap autoimmune disease rather than “pure” SSc [8]. Despite these insights, the clinical meaning of routinely available serum complement parameters in SSc remains incompletely defined.

Most prior studies in SSc have focused on total C3 and C4 concentrations or on specialized activation products such as C4d or terminal complement complex, often within narrowly defined clinical subsets [9]. Biologically, C3c represents an activation-related degradation product of C3 that is generated during complement activation and is widely measured in routine laboratories using standardized immunonephelometric assays. Conceptually, C3c may provide a more direct readout of ongoing complement activation than total C3, which reflects the balance between hepatic synthesis and consumption. Nevertheless, despite its broad availability and established use in other autoimmune settings, the role of serum C3c in SSc has not been systematically explored [10].

Despite increasing evidence of complement involvement in systemic sclerosis, the clinical meaning of routinely available serum C3c, as reported in routine laboratory practice, has not been defined. To our knowledge, C3c specifically has not been previously evaluated in SSc in relation to acute-phase reactants, organ phenotype, extended autoantibody profiles, immunosuppressive treatment, and patient-reported outcomes collected within a unified clinical framework. As a result, it remains unclear whether serum C3c primarily reflects systemic inflammation, organ-specific disease manifestations, immunological phenotype, or patient-perceived disease burden in SSc.

Addressing this gap is clinically relevant for several reasons. First, inflammatory markers such as interleukin-6 (IL-6), C-reactive protein (CRP), and erythrocyte sedimentation rate (ESR) are imperfect indicators of disease activity in SSc and show variable associations with clinical outcomes [1,2]. Second, complement-directed therapies are entering routine rheumatology practice in other systemic autoimmune diseases, increasing the need to understand how complement biomarkers behave across different disease contexts [5]. Finally, clarifying the interpretative value of serum C3c may help align mechanistic insights into complement activation with pragmatic clinical assessment in SSc.

Therefore, the aim of this study was to systematically evaluate serum C3c and C4 in a well-characterized cohort of patients with systemic sclerosis, in relation to systemic inflammation, detailed clinical phenotypes (including skin involvement, interstitial lung disease, and gastrointestinal involvement), immunological profiles, immunosuppressive therapy, and validated patient-reported outcomes. By embedding complement measurements into a multidimensional, same-day clinical assessment, we sought to define the clinical signal domain of serum C3c in SSc and to clarify how routine complement testing should be interpreted in contemporary clinical practice and research.

## 2. Results

### 2.1. Cohort Characteristics

Seventy patients with systemic sclerosis (SSc) were included (Table 1). The cohort was predominantly female (84.3%), with a median age of 57.0 years and median disease duration of 4.0 years. Interstitial lung disease (ILD) was present in 37.7% of evaluable patients, gastrointestinal involvement in 24.6%, and pulmonary hypertension in 5.8%. Autoantibody distribution reflected typical SSc patterns, with Scl-70 and ACA detected in 48.4% and 51.6%, respectively. The median mRSS was 6.0. Patient-reported outcomes indicated moderate disease burden: median PGA was 51.5 mm, VAS pain 50.0 mm, HAQ-DI 0.75, and SScQoL 14.0 points. Current immunosuppressive therapy included MMF (45.7%), MTX (41.4%), and AZA (5.7%); 10.0% had received CYC in the past.

**Figure 1:**
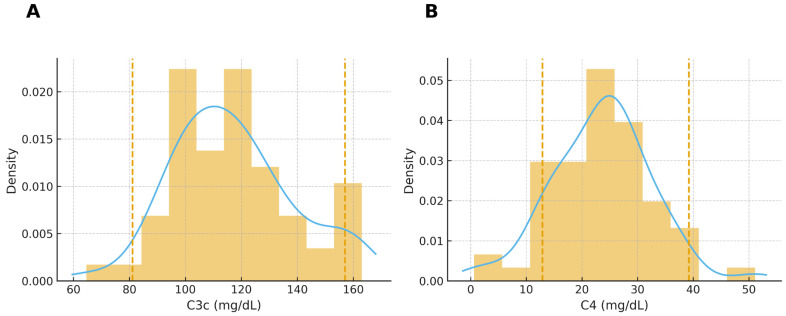
Distribution of serum complement C3c and C4 in the study cohort. (**A**) Histogram of serum C3c concentrations. (**B**) Histogram of serum C4 concentrations. Bars represent density-normalized histograms, and the blue curve represents the kernel density estimate of the distribution. Orange dashed vertical lines indicate the lower and upper laboratory reference limits for each analyte (C3c: 81.1–157.0 mg/dL; C4: 12.9–39.2 mg/dL). The *y*-axis (“Density”) represents probability density.

Median serum complement levels were within reference ranges: C3c 115.7 mg/dL and C4 24.7 mg/dL. The distribution of C3c and C4 values is illustrated in Figure 1.

### 2.2. Associations Between Complement Components and Systemic Inflammation

**Figure 2:**
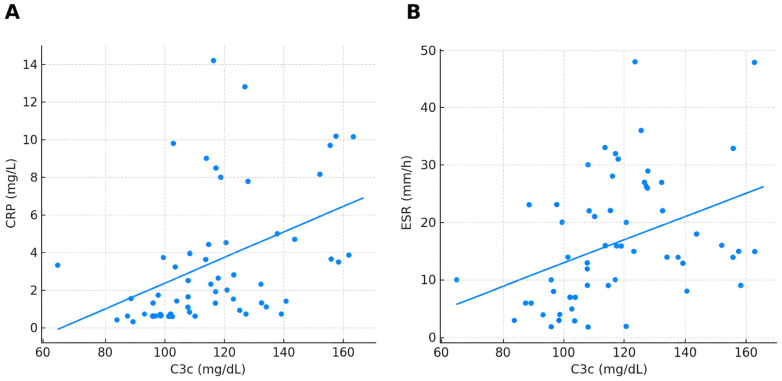
Associations between serum C3c and systemic inflammatory markers in systemic sclerosis. (**A**) Scatter plot showing the association between serum C3c and CRP. (**B**) Scatter plot showing the association between serum C3c and ESR. Each marker represents one patient. Solid lines indicate the fitted linear regression trend.

C3c showed strong, highly significant associations with acute-phase reactants. C3c correlated positively with CRP (ρ = 0.53, *p* < 0.001) and ESR (ρ = 0.49, *p* < 0.001) (Table 2), and these associations remained independent in multivariable linear regression adjusted for age, sex, and disease duration. C3c independently predicted CRP (β = 0.26 mg/L per mg/dL; 95% CI 0.14–0.38; *p* < 0.001; R^2^ = 0.17) and ESR (β = 0.32 mm/h per mg/dL; 95% CI 0.16–0.47; *p* < 0.001; R^2^ = 0.15). Scatterplots illustrating these relationships are shown in Figure 2.

C4 demonstrated weaker but statistically significant correlations with CRP (ρ = 0.37, *p* = 0.004) and ESR (ρ = 0.29, *p* = 0.03).

Importantly, neither C3c nor C4 correlated with IL-6 (all q > 0.10).

### 2.3. Complement Components and Clinical or Serological Phenotypes

The negative phenotype analyses are presented below by clinical, serological, patient-reported, and treatment-related domains. Complement levels did not differ between diffuse and limited cutaneous subsets, nor between patients with and without ILD, gastrointestinal involvement, pulmonary hypertension, or abnormal mRSS (Table 3). C3c and C4 showed no associations with any SSc-specific autoantibodies, including ACA, Scl-70, PM/Scl, Ku, Th/To, RP11, RP155, Ro-52, or NOR-90.

Similarly, neither C3c nor C4 showed associations with patient-reported outcomes (PGA, VAS pain, HAQ-DI, or SScQoL), and there were no differences in complement levels according to MMF, MTX, AZA, or past CYC therapy. All exploratory associations were non-significant after FDR correction.

### 2.4. Hypocomplementemia and Overlap Autoimmune Disease

Hypocomplementemia (C3c < 81.1 mg/dL and/or C4 < 12.9 mg/dL) was rare, identified in 5 of 70 patients (7.1%) (Table 4). Notably, 4 of these 5 patients (80%) had defined overlap autoimmune disease, most commonly secondary SjD or autoimmune thyroiditis. In contrast, hypocomplementemia was infrequent in “pure” SSc without overlap features. No hypocomplementemic patient had diffuse or progressive cutaneous involvement.

**Figure 3:**
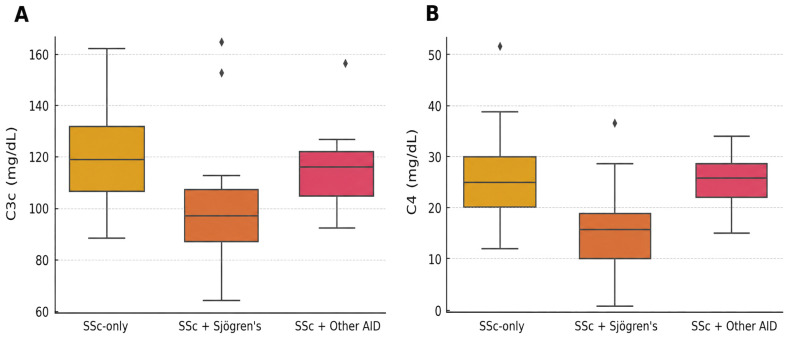
Serum C3c and C4 levels according to autoimmune overlap group. (**A**) Box plot of serum C3c levels across autoimmune overlap groups. (**B**) Box plot of serum C4 levels across autoimmune overlap groups. Boxes represent the interquartile range (IQR), the central horizontal line indicates the median, whiskers extend to the most extreme values within 1.5 × IQR, and points beyond the whiskers represent outliers. AID—autoimmune diseases.

In an exploratory focused subgroup analysis, serum complement levels were compared between patients with and without overlap autoimmune disease (Figure 3). C3c and C4 concentrations were significantly lower in patients with secondary SjD (*n* = 11) compared to those with SSc-only: C3c median 97.7 [IQR 20.2] vs. 119.6 mg/dL (*p* = 0.0129, q = 0.039); C4 median 15.7 [IQR 9.0] vs. 24.9 mg/dL (*p* = 0.0041, q = 0.026). No such differences were observed in patients with autoimmune thyroiditis or other overlap autoimmune diseases.

## 3. Discussion

To our knowledge, this is the first study to systematically evaluate complement fragment C3c specifically in systemic sclerosis. Our findings are particularly relevant given the widespread availability of serum C3c testing as a marker of complement activation in routine diagnostic laboratories worldwide. This accessible readout, commonly embedded in standard complement panels, has until now lacked systematic evaluation in the context of SSc.

Complement activation has long been implicated in systemic sclerosis (SSc), yet the clinical interpretation of routinely measured serum complement parameters remains incompletely defined. Early studies demonstrated systemic complement activation in SSc and suggested associations with disease severity [6], while subsequent work identified distinct complement biomarker patterns in selected high-risk manifestations such as scleroderma renal crisis [7] and fibrotic skin and lung involvement [8]. Large multicentre data further showed that true hypocomplementemia is uncommon in SSc and is more frequently associated with overlap autoimmune phenotypes than with “pure” SSc itself [9,11]. Despite these advances, an important translational gap persisted: the role of serum C3c—an activation-related complement fragment available in routine diagnostics—has not been systematically evaluated in SSc within a multidimensional clinical framework integrating inflammation, clinical phenotype, immunological profile, treatment exposure, and patient-reported outcomes (PROs).

The principal finding of this study is that serum C3c is robustly and independently aligned with the acute-phase response in SSc, showing strong associations with both CRP and ESR after adjustment for age, sex, and disease duration. To our knowledge, this represents the first systematic demonstration in SSc that an activation-linked complement readout behaves in concert with established inflammatory markers. This observation provides a clinically interpretable anchor for complement testing in SSc and complements—rather than duplicates—the established, yet imperfect, roles of CRP and ESR in disease assessment [1,3]. However, these cross-sectional data do not establish that C3c improves clinical decision-making beyond CRP or ESR. In this regard, our data help bridge a gap between mechanistic evidence of complement involvement in SSc [6,7,8] and the day-to-day uncertainty surrounding how serum complement results should be interpreted in clinical practice.

A key interpretative aspect concerns the distinction between C3c and total C3. Although total C3 is more commonly used in clinical laboratories, it was not measured in this cohort. The study was designed to focus on C3c as an activation-related degradation product, expected to more directly reflect dynamic complement engagement than total C3, which may be less responsive to short-term fluctuations in immune activation. Total C3 reflects the balance between hepatic synthesis and consumption, whereas C3c represents an activation-related degradation product and is therefore conceptually closer to ongoing complement engagement. In SSc, where complement activation has been implicated in vascular injury and tissue remodeling [6,7,8], an activation-linked marker would plausibly track inflammatory biology. The tight coupling of C3c with CRP and ESR observed in our cohort supports the interpretation that, in an unselected SSc population, serum C3c primarily reflects systemic inflammatory activity rather than organ-specific fibrotic burden. This is consistent with broader evidence that inflammatory markers in SSc, while informative at the population level, do not straightforwardly map onto individual organ involvement in cross-sectional analyses [1,2,3,4]. Importantly, we did not observe any association of C3c with IL-6, suggesting that C3c and IL-6 capture partially distinct dimensions of inflammatory biology in SSc, at least in a cross-sectional inpatient setting.

Conceptually and analytically, serum C4 conveyed more limited incremental information. Although nominal correlations with CRP and ESR were observed, these signals were less robust within the multiplicity-aware exploratory framework. This does not question the biological relevance of the classical complement pathway in SSc; rather, it indicates that routine serum C4 provides a less sensitive correlate of inflammatory variation than C3c in this cohort. Prior studies similarly suggest that complement dysregulation in SSc may be most apparent in specific clinical contexts or through selected complement biomarkers, while global serum measures frequently remain within reference ranges in unselected cohorts [7,8,9].

Importantly, neither C3c nor C4 stratified clinical phenotype, autoantibody profile, or patient-perceived disease burden. Despite comprehensive phenotyping—including cutaneous subsets, ILD, gastrointestinal involvement, pulmonary hypertension probability, mRSS, and an extended autoantibody panel—complement levels did not differentiate organ manifestations or skin involvement, and did not relate to PGA, pain, HAQ-DI, or SScQoL. Rather than suggesting lack of biological relevance, these findings define the clinical signal domain of routinely measured serum complement in SSc: robust for systemic inflammatory tone, but not for cross-sectional phenotype discrimination or PRO burden. This should be interpreted within the broader multidimensional biomarker landscape of SSc, in which inflammatory, vascular, microvascular, capillaroscopic, ocular, and organ-specific assessment tools may capture partially dissociated disease domains [4,5,11,12,13].

The observed associations were not explained by immunosuppressive therapy. Complement levels did not differ according to current MMF, MTX, or AZA use, nor according to prior cyclophosphamide exposure, and the relationship between C3c and inflammatory markers remained independent of major clinical confounders. This supports the interpretation that the observed C3c–inflammation signal reflects intrinsic inflammatory biology rather than treatment-related effects in this cohort.

Hypocomplementemia was rare and occurred predominantly in patients with overlap autoimmune disease, most commonly secondary SjD or autoimmune thyroiditis. This reproduces multicentre EUSTAR observations [9] and provides a clinically constructive message: low complement levels in SSc should prompt targeted evaluation for overlap autoimmune disease rather than being interpreted as a marker of aggressive “pure” SSc. In other systemic autoimmune diseases—particularly systemic lupus erythematosus and SjD—low complement is a routine marker of immune complex–mediated activity and monitoring [14,15]. Our data refine the position of SSc within this broader rheumatology landscape: complement activation is biologically relevant [6,7,8], but routine serum complement readouts in unselected SSc patients behave predominantly as markers of systemic inflammatory milieu and as flags for overlap autoimmunity when truly low [9,14].

Importantly, in this exploratory analysis, among patients with overlap autoimmune diseases, those with secondary SjD exhibited a distinct profile characterized by significantly lower serum C3c and C4 levels. This observation supports the interpretation that true complement consumption in SSc is more tightly linked to immune complex–mediated overlap syndromes than to SSc itself. The absence of similar findings in autoimmune thyroiditis or other overlap types further refines the clinical signal domain of complement biomarkers and underscores the diagnostic specificity of Sjögren-associated complement alterations.

Several methodological features strengthen the validity of these findings, including prospective and consecutive enrolment, same-day acquisition of clinical, laboratory, and PRO data, centralized complement measurement with defined reference ranges, and a statistical strategy that separated predefined hypotheses from exploratory analyses with FDR control.

This study has several limitations. First, it was a single-center, cross-sectional analysis with a moderate sample size of 70 patients, which limits causal inference, external generalizability, and assessment of longitudinal changes in complement parameters. Second, the study evaluated routinely measured serum C3c and C4, but did not include total C3 or dedicated complement activation products such as C3a, C3d, C4d, or terminal complement complex. Third, subgroup analyses, particularly those involving hypocomplementemia and secondary SjD, were based on small numbers and should therefore be interpreted as exploratory. Finally, the absence of associations with clinical phenotype, organ involvement, treatment exposure, or PROs should be interpreted in the context of the exploratory design and limited statistical power for some subgroup comparisons.

Taken together, this study advances the complement narrative in SSc by providing the first multidimensional evaluation of serum C3c in relation to inflammation, phenotype, serology, treatment, and patient-reported outcomes. By defining where the clinical signal of C3c is strongest—and where it is not—our findings close an important translational gap between complement biology in SSc and the interpretation of routine serum complement testing in contemporary practice and research.

## 4. Materials and Methods

### 4.1. Study Population

This was a cross-sectional exploratory study based on a prospectively enrolled cohort of 70 consecutive patients with systemic sclerosis (SSc) fulfilling the 2013 American College of Rheumatology/European League Against Rheumatism (ACR/EULAR) classification criteria [16]. All patients were recruited at the Department of Rheumatology, Connective Tissue Diseases, and Rare Diseases, National Medical Institute of the Ministry of the Interior and Administration in Warsaw, Poland, between February 2024 and November 2025.

Exclusion criteria were: localized scleroderma, sine scleroderma, significant renal impairment (Cockcroft–Gault creatinine clearance <45 mL/min), clinically significant hepatic insufficiency, New York Heart Association class III/IV heart failure, or hematologic disease.

All patients provided written informed consent. The study was approved by the institutional ethics committee and conducted in accordance with the Declaration of Helsinki.

### 4.2. Clinical and Laboratory Assessments

Skin involvement was assessed using the modified Rodnan skin score (mRSS) [17]. Interstitial lung disease (ILD) was defined as fibrotic abnormalities on high-resolution computed tomography (HRCT) [18]. 

Gastrointestinal involvement was defined by typical symptoms (dysphagia, reflux, malabsorption) and/or confirmatory endoscopy/radiography.

Pulmonary hypertension was defined based on echocardiographic probability according to contemporary guidelines.

Autoimmune overlap included secondary SjD, autoimmune thyroiditis or other defined organ specific overlap autoimmune disease.

Systemic inflammation was assessed using erythrocyte sedimentation rate (ESR), C-reactive protein (CRP), and interleukin-6 (IL-6).

The autoantibody panel included anti-centromere (ACA), anti-topoisomerase I (Scl-70), and a broad set of SSc-associated antibodies (PM/Scl75, PM/Scl100, Ku, Th/To, Ro-52, NOR-90, RP11, RP155), determined using EUROLINE immunoblotting (EUROIMMUN Medizinische Labordiagnostika AG, Lübeck, Germany).

Patient-reported outcomes (PROs) were collected on the same day as clinical assessment and blood sampling, using:

Visual Analog Scale (VAS) for patient—reported global disease activity (PGA),
VAS for pain,
Health Assessment Questionnaire Disability Index (HAQ-DI) [19,20],
the Polish version of the Systemic Sclerosis Quality of Life Questionnaire (SScQoL) [21].

Serum complement C3c and C4 were measured in a single central laboratory using particle-enhanced nephelometric immunoassay (PENIA) on the BN II System (Siemens Healthcare Diagnostics Products GmbH, Marburg, Germany), according to the manufacturer’s protocol. Results were expressed in mg/dL. Reference ranges were: C3c 81.1–157.0 mg/dL, C4 12.9–39.2 mg/dL. Hypocomplementemia was defined as C3c < 81.1 mg/dL and/or C4 < 12.9 mg/dL. For clarity, the term C3c in this manuscript denotes the routine serum complement result reported by the central laboratory and was interpreted in its clinical laboratory context, not as an isolated measurement of free C3c or other dedicated complement activation fragments.

Immunosuppressive therapy was recorded as current use of mycophenolate mofetil (MMF), methotrexate (MTX), or azathioprine (AZA), and past or current use of cyclophosphamide (CYC). No patient was receiving CYC within 6 months prior to blood sampling.

### 4.3. Bias Minimisation

To minimise selection bias, all eligible patients were consecutively enrolled. Clinical assessment, PROs, and laboratory testing were performed during the same inpatient visit to ensure temporal alignment. The order of assessments was standardized.

mRSS scoring was performed by trained rheumatologists calibrated before study initiation to reduce inter-observer variability [17]. PRO questionnaires were administered by trained staff blinded to laboratory results.

All assays were performed in one central laboratory using identical protocols. Data were independently double-entered and cross-checked for accuracy.

### 4.4. Study Size

No a priori sample size calculation was performed given the exploratory nature of the biomarker analysis. The final cohort size reflects all eligible patients with available complement measurements. Analyses involving small subgroups (e.g., hypocomplementemia *n* = 5) were interpreted cautiously as exploratory.

### 4.5. Statistical Analysis

Continuous variables were summarised as median (interquartile range, IQR) and categorical variables as number (%). Between-group differences were analysed using the Mann–Whitney U test (continuous variables) and χ^2^ or Fisher’s exact tests (categorical variables).

Associations between complement components (C3c, C4) and continuous measures were assessed using Spearman’s rank correlation (ρ). CRP, ESR, and IL-6 were assessed as inflammatory markers. For inflammatory markers showing significant univariable associations with complement components, multivariable linear regression adjusted for age, sex and disease duration was used to evaluate independent associations; model coefficients (β), 95% confidence intervals (CIs) and explained variance (R^2^) are reported.

Given the limited number of complement-related primary hypotheses and a strong a priori expectation regarding associations with CRP and ESR, these analyses were not subject to multiplicity correction. For exploratory comparisons (complement vs. PROs, organ involvement, autoantibodies, or overlap autoimmune diseases), *p*-values were corrected using the Benjamini–Hochberg false-discovery rate (FDR) procedure [22,23].

The association between hypocomplementemia and overlap autoimmune disease was analysed using Fisher’s exact test and reported with nominal *p*-values, because this biologically predefined comparison was not part of a family of independent tests.

All analyses were complete-case; no imputation was performed. Statistical significance was defined as *p* < 0.05 for primary analyses and q < 0.10 for FDR-adjusted exploratory analyses [22,23,24]. Analyses were performed using GraphPad Prism v9.0 (GraphPad Software, Boston, MA, USA) and IBM SPSS Statistics v28.0 (IBM Corp., Armonk, NY, USA).

IL-6 was excluded from multivariable models as it did not show a statistically significant correlation with either C3c or C4 (q > 0.10), suggesting a distinct inflammatory axis not closely aligned with complement activation in this cohort.

## 5. Conclusions

This study provides the first systematic evidence in systemic sclerosis that serum C3c—a test widely available in routine diagnostics—is robustly and independently aligned with CRP and ESR, defining C3c as an accessible marker of systemic inflammatory response in SSc. C4 contributed more limited incremental information, and neither complement measure captured clinical phenotype, organ involvement, extended autoantibody profile, treatment status, or patient-reported burden in an unselected cohort. True hypocomplementemia was rare and occurred predominantly in overlap autoimmune disease, supporting its value as a clinical indicator of concomitant immune complex–mediated autoimmunity. Together, these findings translate mechanistic evidence of complement involvement in SSc into a clinically interpretable serum framework and close an important gap in how routine complement testing should be understood in SSc care and research.

## Figures and Tables

**Table 1:** Cohort characteristics.

Variable	Value
**Demographic and disease characteristics**	
Number of patients, *n*	70
Female sex, *n* (%)	59 (84.3)
Age, years, median [IQR]	57.0 [48.0–67.0]
Disease duration, years, median [IQR]	4.0 [2.0–10.0]
**Clinical phenotype and organ involvement**	
Interstitial lung disease (HRCT), *n*/*N* (%)	26/69 (37.7)
Gastrointestinal involvement, *n*/*N* (%)	17/69 (24.6)
Pulmonary hypertension, *n*/*N* (%)	4/69 (5.8)
mRSS, points, median [IQR]	6.0 [2.0–8.75]
Scl-70–positive, *n*/*N* (%)	31/64 (48.4)
ACA-positive, *n*/*N* (%)	33/64 (51.6)
Coexisting autoimmune disease, *n*/*N* (%)	25/70 (35.7)
**Inflammation and complement**	
ESR, mm/h, median [IQR]	15.0 [8.0–30.0]
CRP, mg/L, median [IQR]	2.3 [0.7–5.1]
IL-6, pg/mL, median [IQR]	3.9 [2.3–6.5]
C3c, mg/dL, median [IQR]	115.7 [102.1–127.6]
C4, mg/dL, median [IQR]	24.7 [17.4–28.6]
Hypocomplementemia *n*/*N* (%)	5/70 (7.1)
Hypocomplementemia with overlap disease, *n*/*N* (%)	4/5 (80.0)
**Immunosuppressive therapy**	
Mycophenolate mofetil *n*/*N* (%)	32/70 (45.7)
Methotrexate *n*/*N* (%)	29/70 (41.4)
Azathioprine *n*/*N* (%)	4/70 (5.7)
Cyclophosphamide (ever; none within last 6 months), *n*/*N* (%)	7/70 (10.0)
**Patient-reported outcomes**	
PGA (VAS global activity), 0–100, median [IQR] (*n* = 70)	51.5 [33.5–69.0]
VAS pain, 0–100, median [IQR] (*n* = 70)	50.0 [26.0–73.8]
HAQ-DI, 0–3, median [IQR] (*n* = 70)	0.75 [0.25–1.25]
SScQoL, 0–29, median [IQR] (*n* = 70)	14.0 [9.25–20.0]

**Table 2:** Associations of C3c and C4 with CRP and ESR.

Variable	Correlation (ρ)	*p*-Value	FDR (q)	Linear Regression (β, 95% CI), R^2^
C3c vs. CRP	0.53	<0.001	<0.001	β = 0.26 (0.14–0.38), R^2^ = 0.17
C3c vs. ESR	0.49	<0.001	<0.001	β = 0.32 (0.16–0.47), R^2^ = 0.15
C4 vs. CRP	0.37	0.004	0.006	β = 0.18 (0.06–0.30), R^2^ = 0.11
C4 vs. ESR	0.29	0.03	0.03	β = 0.21 (0.02–0.39), R^2^ = 0.08

**Table 3:** Serum C3c and C4 across clinical phenotypes in systemic sclerosis.

Phenotype	C3c, Median [IQR] (mg/dL)	*p*-Value	C4, Median [IQR] (mg/dL)	*p*-Value
Diffuse vs. limited SSc	114.9 [101.8–126.2] vs. 116.1 [102.5–128.4]	0.89	24.1 [17.0–28.4] vs. 24.9 [17.7–28.8]	0.77
ILD vs. no ILD	115.0 [102.0–126.0] vs. 116.3 [102.6–128.8]	0.95	24.0 [17.2–28.0] vs. 24.9 [17.6–28.8]	0.81
GI involvement vs. none	115.5 [101.9–127.3] vs. 116.0 [102.3–128.2]	0.92	24.3 [17.1–28.5] vs. 24.8 [17.8–28.6]	0.84

**Table 4:** Hypocomplementemia and Overlap Autoimmune Disease.

Variable	Value
Hypocomplementemia (C3c < 81.1 or C4 < 12.9 mg/dL), *n*/*N* (%)	5/70 (7.1%)
Overlap autoimmune disease among hypocomplementemic patients, *n*/*N* (%)	4/5 (80.0%)

## Data Availability

All data analysed or generated during this study are included in this published article. Further de-identified data sets and analytical scripts are available from the corresponding author upon reasonable request for academic and non-commercial purposes.
